# Fatal Community-Acquired Clostridioides difficile Infection as a Cause of Listeria Meningitis: A Case Report

**DOI:** 10.7759/cureus.54371

**Published:** 2024-02-17

**Authors:** Kai Naraoka, Hajime Ikenouchi, Tatsuo Miyamoto, Kensho Ikeda, Kaoru Endo

**Affiliations:** 1 Neurology, Sendai City Hospital, Sendai, JPN

**Keywords:** fatal case, clostridioides difficile infection, hypoalbuminemia, infectious colitis, listeria meningitis

## Abstract

A 77-year-old woman with a history of total gastrectomy was transferred to our hospital with complaints of fever and consciousness disturbance for five days. She had fever and consciousness disturbance with positive meningeal signs. Laboratory findings indicated an elevated inflammatory response and hypoalbuminemia, and computed tomography (CT) of the body indicated intestinal gas retention and mild ascites. Cerebrospinal fluid analysis revealed pleocytosis with elevated protein levels and a diagnosis of Listeria meningitis was made. Treatment with ampicillin/sulbactam was started, and her fever and consciousness disturbance resolved on day 2. However, on day 3, her fever and conscious disturbance deteriorated, and she went into shock subsequently. Laboratory findings revealed deteriorated inflammatory response and hypoalbuminemia. Body CT showed an obvious distended bowel loop and intestinal edema. A stool culture revealed positive Clostridioides difficile toxin B, and we diagnosed her with Clostridioides difficile infection (CDI). Although intravenous metronidazole was initiated, she died due to prolonged hypovolemic shock. We considered she had community-acquired CDI because her CDI emerged immediately after the initiation of antibiotics, symptom deterioration within 48 hours of admission, and abnormal abdominal CT findings at admission. Listeria meningitis can develop based on community-acquired CDI. Because CDI can have a very rapid and fatal course and is sometimes complicated by other infectious diseases, clinicians should pay attention to this complication.

## Introduction

Clostridioides difficile infection (CDI) often presents with watery diarrhea and usually occurs during hospitalization or in patients previously treated with antibiotics [1]. Its diagnosis is sometimes challenging because of its variable presentation, including cases without diarrhea or community-acquired cases within symptom onset 48 hours of admission (CA-CDI) [1,2]. CDI is also reported to be associated with a variety of severe infections transmitted from the intestinal tract [3]. Listeria monocytogenes is transmitted through the gastrointestinal tract and causes bacterial meningitis as a result of bloodstream infection [4]. Therefore, CDI can have an important role in the development of Listeria infection. However, there have been few reported cases of Listeria meningitis associated with CA-CDI. Here, we report a fatal case of CA-CDI that rapidly progressed to hypovolemic shock early after antibiotic therapy for Listeria meningitis.

## Case presentation

**Figure 1:**
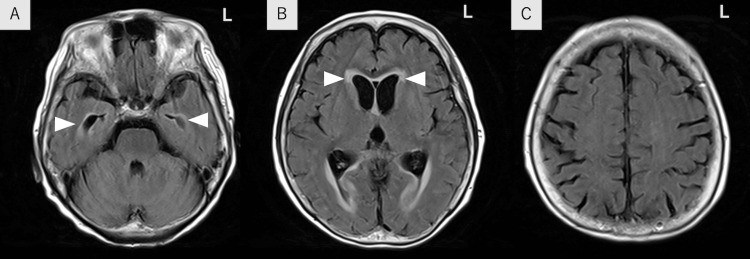
Brain magnetic resonance imaging findings on admission A-C: Brain magnetic resonance imaging showed slight hydrocephalus with periventricular hyperintensity (arrowheads). However, there were no brain lesions in fluid-attenuated inversion recovery.

A 77-year-old woman with a history of total gastrectomy at the age of 57 came to our hospital because of fever and consciousness disturbance over five days. She did not take any medications, including antibiotics before admission. Five days before admission, she had a decreased appetite. She subsequently developed a fever and headache, and her consciousness disturbance gradually progressed. She did not show abdominal symptoms such as diarrhea, abdominal pain, or vomiting. On arrival at the hospital, her vital signs were normal, except for an elevated body temperature of 40.0 °C. Neurological findings indicated a moderate consciousness disturbance with a Glasgow Coma Scale score of 8 (E1V2M5) and a stiff neck. Laboratory findings indicated an elevated white blood cell (WBC) count of 14,400/μL, elevated C-reactive protein (CRP, 9.96 mg/dL), and hypoalbuminemia (2.3 g/dL). Cerebrospinal fluid (CSF) examination revealed elevated pleocytosis (350 cells/μL, with 58% mono-morphonuclear leukocytes), and elevated protein (309 mg/dL). A FilmArray Meningitis/Encephalitis Panel test, which can rapidly test for a wide range of bacteria and viruses in the CSF [5], was positive for Listeria monocytogenes. Two sets of blood cultures also revealed Listeria monocytogenes. Brain magnetic resonance imaging (MRI) revealed slight hydrocephalus with periventricular hyperintensity, and no lesions were observed in the brainstem or cerebral parenchyma (Figures 1A-1C).

**Figure 2:**
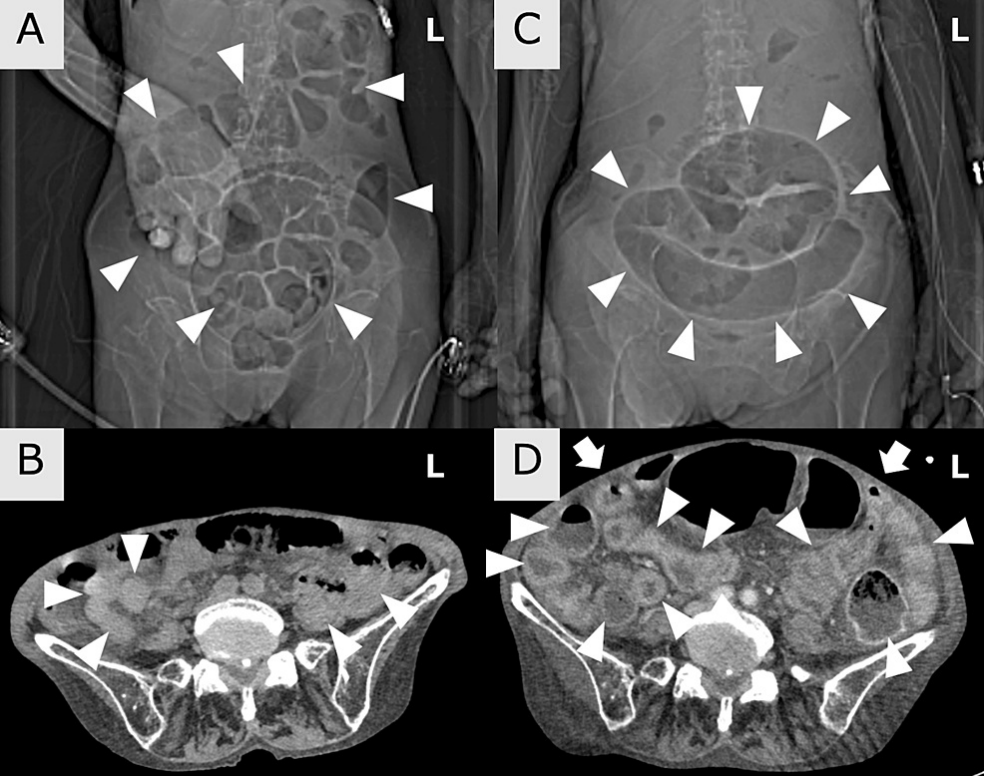
Imaging findings of this case A: On admission (day 1), a scout view of body computed tomography (CT) showed intestinal gas retention and a slightly distended bowel loop (arrowheads); B: Non-contrast-enhanced body CT showed mild intestinal edema (arrowheads); C: On day 5 after symptom deterioration, a scout view of the body CT showed an obvious distended bowel loop like coffee-bean sign (arrowheads); D: Contrast-enhanced body CT showed a distended abdomen (arrows) and intestinal edema (arrowheads).

A scout view of computed tomography (CT) showed intestinal gas retention and a slightly distended bowel loop (Figure 2A). Non-contrast enhanced CT showed intestinal gas retention and mild intestinal edema (Figure 2B). As the diagnosis was Listeria meningitis, intravenous ampicillin was started. On day 2, her fever and conscious disturbance were markedly resolved. However, on day 3, within 48 hours of admission, the fever, abdominal pain, and consciousness disturbance recurred, and on day 5, she became in a shock state. Her vital signs showed tachycardia of 108 bpm, decreased blood pressure of 82/56 mmHg, and consciousness disturbance of Glasgow Coma Scale (GCS) 12 (E3V4M5). Physical examination showed a distended abdomen and her extremities were cold. Blood tests showed an elevated WBC count (23,400/μL), decreased platelet count (75,000/μL), elevated CRP (14.19 mg/dL), and hypoalbuminemia had progressed (1.2 g/dL). We considered she was in hypovolemic shock or septic shock. A scout view of body CT showed an obvious distended bowel loop-like coffee-bean sign (Figure 2C), and contrast-enhanced body CT showed a distended abdomen and intestinal edema (Figure 2D).

We first suspected sigmoid volvulus, but the colon fiber did not show sigmoid torsion, obstruction, or pseudomembranous enteritis. There was no tumor. Since diarrhea was observed during colon fiber, we consider enteritis. Two sets of blood cultures did not detect Listeria monocytogenes or other causes of enteritis. On the other hand, a stool culture became positive for CD toxin B, and a diagnosis of CDI was made. After treatment with albumin replacement and metronidazole, her WBC and CRP levels gradually improved. However, the hypoalbuminemia did not improve, and the shock state was prolonged. The patient died on day 13.

## Discussion

Clostridioides difficile, a gram-positive, toxin-producing bacteria, is a common cause of nosocomial infections and hospitalization. Although diarrhea is a typical symptom of CDI, some cases do not develop diarrhea but present with other symptoms or imaging findings including ileus, megacolon, distended bowel loops, and fulminant colitis. Laboratory findings could show leukocytosis, elevated CRP, and hypoalbuminemia [1]. Therefore, the diagnosis of CDI is sometimes challenging in atypical cases. In this case, the patient had elevated WBC, CRP, hypoalbuminemia, distended bowel loops, and intestinal edema, which deteriorated after successful treatment for Listeria meningitis. These symptoms aligned with the clinical presentation of CDI [1]. In addition, these suggestive findings of CDI were present at admission in the retrospective view and symptom deterioration occurred within 48 hours after admission. Therefore, this case would have CA-CDI.

The rapid CDI progression after antibiotic initiation was unusual in the present case. Antibiotics may alter the intestinal microbiota and increase the risk of CDI [6]. Symptom deterioration early after antibiotic initiation would be derived from intestinal microbiota change before admission. Gastrectomy decreases gastric acid secretion and alters the intestinal flora, increasing the risk of CDI [1,7], which may have been a distant cause of the onset of CA-CDI and the early symptom deterioration after antibiotics initiation.

Listeria is an intracellular parasite that infects adults by invading the intestinal mucosa after oral intake [4]. In a previous case of Listeria meningitis after CDI in a non-immunocompromised individual, the onset of Listeria meningitis was slower than in the present case [3]. This case reported CD-induced inflammation impaired intestinal mucosal barrier functions, resulting in Listeria meningitis. Although the pathophysiological mechanism could not be confirmed, we speculated a similar mechanism would occur in the present case.

There have been no cases of CA-CDI worsening immediately after antibiotic treatment for Listeria meningitis. This case highlights the importance of considering CA-CDI as a cause of Listeria meningitis. Since CDI can cause rapid and fatal course, clinicians should pay attention to CA-CDI as a cause of rapid abdominal symptom deterioration after antibiotics as well as the cause of other infections derived from the intestinal tract.

## Conclusions

This case demonstrates that CA-CDI can be a cause of Listeria meningitis, with an emphasis on rapid symptom deterioration and fatality after treatment for Listeria meningitis. The speculated mechanism described here might apply to all infections originating from the intestinal tract. In severe infections, clinicians should consider the coexistence of CA-CDI and rapid symptom deterioration during treatment.

## References

[1] Czepiel J, Dróżdż M, Pituch H (2019). Clostridium difficile infection: review. Eur J Clin Microbiol Infect Dis.

[2] Ofori E, Ramai D, Dhawan M, Mustafa F, Gasperino J, Reddy M (2018). Community-acquired Clostridium difficile: epidemiology, ribotype, risk factors, hospital and intensive care unit outcomes, and current and emerging therapies. J Hosp Infect.

[3] Carannante N, Pagliano P, Rossi M (2017). Invasive listeriosis in a patient with several episodes of antibiotic associated colitis presumably due to Clostridium difficile. Infection.

[4] Quereda JJ, Morón-García A, Palacios-Gorba C, Dessaux C, García-Del Portillo F, Pucciarelli MG, Ortega AD (2021). Pathogenicity and virulence of Listeria monocytogenes: a trip from environmental to medical microbiology. Virulence.

[5] Trujillo-Gómez J, Tsokani S, Arango-Ferreira C (2022). Biofire FilmArray Meningitis/Encephalitis panel for the aetiological diagnosis of central nervous system infections: a systematic review and diagnostic test accuracy meta-analysis. EClinicalMedicine.

[6] Theriot CM, Bowman AA, Young VB (2016). Antibiotic-induced alterations of the gut microbiota alter secondary bile acid production and allow for clostridium difficile spore germination and outgrowth in the large intestine. mSphere.

[7] Maksimaityte V, Bausys A, Kryzauskas M (2021). Gastrectomy impact on the gut microbiome in patients with gastric cancer: a comprehensive review. World J Gastrointest Surg.

